# Validation of a questionnaire for assessing fecal impaction in the elderly: impact of cognitive impairment, and using a proxy

**DOI:** 10.1186/1471-2318-13-24

**Published:** 2013-03-07

**Authors:** Marta Barcelo, Maria Jose Jimenez-Cebrian, Manuel Diaz-Rubio, Alberto Lopez Rocha, Enrique Rey

**Affiliations:** 1 Functional GI DiseasesUnit. Division of DigestiveDiseases, Hospital Clínico San Carlos, Departament of Medicine, Universidad Complutense de Madrid, Instituto de Investigación Sanitaria del Hospital Clínico San Carlos (IdISSC), Madrid, Spain; 2 “Valdeluz” Nursing Homes, Madrid, Spain; 3 Spanish Society of Nursing Homes’ Physicians (SEMER), Madrid, Spain

## Abstract

**Background:**

Studies on the epidemiology of fecal impaction are limited by the absence of a valid and reliable instrument to identify the condition in the elderly. Our aim is to validate a questionnaire for identifying fecal impaction in the elderly and to assess the impact of cognitive impairment and the aid of a proxy on its reliability.

**Methods:**

We developed a 5 questions’ questionnaire. The questionnaire was presented to twenty doctors to test its face validity. Feasibility was pre-tested with ten non institutionalized subjects who completed the questionnaire twice, once alone or with the help of a proxy, and another along with the researcher.

For the validation of the questionnaire all residents in a single nursing-home were invited to participate, allowing the self-decision of using a proxy. Medical records of all subjects were abstracted without knowledge of subjects’ answers and agreement between fecal impaction according to self-reported and medical records analyzed. Physical impairment was measured with the Barthel’s test and cognitive impairment with the mini-mental test.

**Results:**

In the face validity only minor changes in wording were suggested. In the feasibility pre-test all subjects were able to understand and complete the questionnaire and all questions were considered appropriate and easily understandable.

One-hundred and ninety-nine of the 244 residents participated in the study (mean age 86,1 ± 6,6). One hundred and forty two subjects understood all questions; not understanding them was inversely associated with cognitive impairment score (aOR: 0.86; 95% CI: 0.82-0.91). One hundred and sixty decided to use a proxy; the use of a proxy was inversely associated with educative level (0.13 (0.02-0.72), minimental’s score (0.85; 0.76-0.95) and Barthel’s score (0.96; 0.94-0.99). Agreement between medical records and self-completed questionnaire was 85.9% (kappa 0.72 (0,62- 0,82). Disagreement was unrelated to education and cognitive impairment.

**Conclusions:**

Our simple questionnaire is reliable for identifying fecal impaction in the elderly by self-report. Limitation imposed by cognitive impairment is minimized with the aid of a proxy.

## Background

Survey research among older adults is becoming increasingly important because of the increase in life expectancy, but faces many challenges. The reliability of self-reported information by the elderly raises some concerns because of their limited ability to provide information [1]. A decrease in cognitive ability is associated with aging [2] and interacts with question’s difficulty to determine the completeness and accuracy of responses in the elderly [3].

The reliability of relatively objective self-reported information, like diagnoses, by the elderly is fairly good [4,5] and this is only slightly reduced by cognitive impairment [6]. However, the reliability of more subjective self-reported information in the elderly is questionable. For example, studies on the applicability of a subjective health outcome measure, such as SF-36, showed that age, cognitive impairment and physical status decrease the rates of self-completion of the SF-36 [7,8], and that cognitive impairment also affects reliability and validity of the self-reported information [9].

The use of proxy respondents is a resource to overcome these limitations, specifically in the setting of an epidemiological survey [10-12]. Proxies are generally considered a reliable source of information, even to measure very subjective outcomes [13], like physical symptoms [14] and well-being [15].

Faecal impaction occurs more frequently in the elderly [16,17], represents a relevant problem for caregivers, and it is potentially associated with complications [18], specially in patients with more than 80 years of age, or patients with heart or neurological disease [19]. Our knowledge of its epidemiology is limited, partly due to the inherent difficulties associated with conducting surveys in the elderly. However there is indirect evidence that suggest that it is a highly prevalent disorder in elderly institutionalized, as are the presence of fecal impaction in 20% of the institutionalized persons who developed fecal incontinence in a period of nearly a year [20], a prevalence of 25% in institutionalized persons with urinary dysfunction [17] and the existence of fecal impaction in 55% of the subjects with diarrhea in a residence [21].

Not only is a common problem but often not diagnosed and therefore undertreated, as reported by Rodriguez KL et al. which shows a prevalence of faecal impaction of 8.8% in older nursing homes hospice/palliative care patients, of whom 26.4% were undertreated [22].

As a first step towards conducting studies in the elderly on this topic, we aimed to develop and validate a simple questionnaire to identify the occurrence of faecal impaction in the elderly and to assess the extent to which a subject’s cognitive impairment, and the aid of a proxy, impact on its reliability.

## Methods

### Development of the instrument

We developed a five question questionnaire to gather information about self-reported episodes of faecal impaction, their frequency, and therapeutic actions to remove them (manual removal, retrograde lavage –enema-, and anterograde lavage -intensive laxative use), with a recall period of one year, including two questions for evaluating understandability and need of a proxy.

Prior to validation in the desired setting (nursing home), we undertook a face validity and feasibility process. The questionnaire was presented to ten gastroenterologists and ten doctors specifically dedicated to medical care in nursing homes to test its face validity. They were asked to provide their opinion about the questions and their suggestions for improvement. Feasibility was pre-tested with ten non institutionalized subjects older than 80 years-old, without significant cognitive impairment. They were asked to complete the questionnaire and afterwards one of the investigators revisited questions and answers with each patient, obtaining feedback about their ability to understand each question and the appropriateness of the answers given.

### Validation of the questionnaire

The study sample was recruited in a single nursing home (Centro “Valdeluz”, Madrid). The quality of care in this nursing home has been certified by the regional authority (“Madrid excellence” certification) and AENOR (ISO 9001:2000). It has the capacity to accommodate 244 residents. All residents were considered eligible and the only exclusion criteria were the definitive inability to answer the questionnaire even with the aid of a proxy (in the opinion of one investigator), and not providing consent to participate.

Participants were asked to complete the final version of the questionnaire. Two questions were added to recall if they completely understood the questionnaire by themselves and whether they needed the assistance of a proxy to complete it. The subjects themselves decided whether or not a proxy was required, as well as who was asked to be the proxy.

The medical and nursing records of the subjects were abstracted by a physician with no knowledge of subjects’ answers to the questions. Using a structured form, information was collected regarding the subjects’ history of faecal impaction, frequency and methods of removal. In addition, information on physical functional and cognitive impairment was recorded. As part of the clinical protocol, at admission and every 6 months thereafter, all residents underwent a Barthel’s test to evaluate physical function [23,24] and the mini-cognitive exam MEC-35 [25], a Spanish validated version of the Folstein´s mini-mental test [26]. A score of 19 to 23 was classified as mild, a score of 14 to 18 as moderate, and less than 14 as severe cognitive impairment [27].

### Analysis

In the literature, definition of Fecal Impaction is elusive, even in the best review published in the literature [28]. Definition was review by Creason and Sparks [18] and among those definitions provided by the literature, we chose the more restrictive which, was considered appropriate by gastroenterologists and nursing homes’ doctors, according to the first part of the design of the questionnaire. Thus, faecal impaction was defined as an accumulation of hard faeces in the rectum that the subject was unable to evacuate themself, occurring at least once in the last year. Level of education was categorized as either primary (school or just able to read and write) or secondary or higher (including high school, professional education and university education).

Feasibility was evaluated in the institutionalized sample of residents by the subjects self-reporting whether they could, or could not, understand all questions. We analyzed to what extent this led to the use of a proxy, and explored whether age, gender, time in the institution, cognitive impairment and level of education were associated with not understanding all of the questions using a regression model.

For the reproducibility test, twenty-eight subjects that were participating in the concurrent validity study were asked to answer the questionnaire on two occasions, 7–10 days apart. They were selected at random (the first 28 participants) from all participants. Concurrent validity was analyzed comparing the agreement between faecal impaction in the last year, as reported in the questionnaire by the subject, and the history of faecal impaction in the last year according to medical records.

Reproducibility and concurrent validity were analyzed by simple agreement and weighted kappa statistics.

We analyzed the factors associated with the decision to use a proxy, including the impact of cognitive and functional impairment and the understandability of the questionnaire as the key influencing variables, adjusting for age, gender, time in residence, and level of education. In addition, we explored whether using a proxy was associated with disagreement between self-reported faecal impaction in the previous year and medical/nurse diagnosis. This was examined using a logistic regression model, adjusting for age, gender, time in residence, level of education, cognitive impairment and functional abilities (Barthel’s score).

### Ethical aspects

This study was approved by the Ethics Committee of the Hospital Clinico San Carlos (**CEIC**- **Code**: 10/014-E) and all data were treated confidentially by the researchers, according to law 15/99 on protection of personal data.

All participants signed the document of informed consent for participation in the study.

## Results

### Questionnaire development

A five questions’ questionnaire was drafted by the first author and subsequently refined by all authors until the version was considered operational. It was then presented to both gastroenterologist and nursing homes’ doctors, who considered that all questions appropriately collected the information they were intended to; some minor changes in wording were suggested. In the feasibility pre-test all subjects were able to understand and complete the questionnaire; in the face-to-face interviews, all answers were considered appropriate and easily understandable.

### Validation

One hundred and ninety nine of the 244 (81.5%) eligible subjects completed the study. Their mean age was 86.1 ± 6.6 (61–104), 144 (72.4%) were females, and 139 (69.8%) had a primary level of education or less. Mean mini-mental score was 23.0 ± 8.4 (range: 0–35) and mean Barthel’s score was 64.5 ± 28.8 (0–100).

#### Feasibility

One hundred and forty two subjects (71.4%) reported that they understood all questions, and 57 acknowledged that they did not. As shown in Table 1, not understanding all of the questions was associated with cognitive impairment, but not with level of education.

**Table 1:** Factors associated with not understanding all questions

	**Understood (N = 142)**	**Did not understand (N = 57)**	**Univariate OR**	Adjusted OR*
Age	86.0 ± 6.40	86.2 ± 7.24	1.00 (0.96-1.05)	1.00 (0.94-1.06)
Female Gender	97 (68.3%)	47 (82.5%)	2.18 (1.01-4.70)	2.53 (0.99-6.49)
Time at the institution	23.3 ± 23.02	28.1 ± 26.15	1.01 (1.00-1.02)	1.01 (0.99-1.02)
Level of education Secondary or higher	128 (92.8%)	56 (98.2%)	0.23 (0.03-1.83)	0.11 (0.01-1.85)
Cognitive impairment (MEC-35 score)	25.8 ± 6.15	16.0 ± 9.29	0.85 (0.81-0.90)	0.86 (0.82-0.91)
Functional impairment (Barthel’s score)	70.2 ± 25.02	50.2 ± 32.47	0.98 (0.97-0.99)	0.99 (0.98-1.01)

*adjusted by all variables in the table.

One hundred and sixty (80.4%) subjects completed the questionnaire with the aid of a proxy. All subjects who self-reported that they did not understand all of the questions looked for the aid of a proxy. Table 2 shows factors associated with the use of a proxy.

**Table 2:** Factors associated with the use of a proxy (subjects who understood all questions)

	**Not use of a proxy (N = 39)**	**Use a proxy (N = 103)**	**Univariate OR**	Adjusted OR*
Age	85.6 ± 5.9	86.2 ± 6.6	1.01 (0.96-1.07)	1.03 (0.96-1.11)
Female Gender	27 (69.2%)	70 (68%)	0.94 (0.43-2.09)	0.91 (0.36-2.36)
Time at the institution	25.8 ± 25.5	22.4 ± 22.1	0.99 (0.98-1.01)	0.99 (0.97-1.01)
Level of education Secondary or higher	7 (17.9%)	3 (3%)	0.14 (0.03-0.59)	0.13 (0.02-0.72)
Cognitive impairment (MEC-35 score)	28.9 ± 3.6	24.6 ± 6.5	0.84 (0.77-0.93)	0.85 (0.76-0.95)
Functional impairment (Barthel’s score)	84.6 ± 19.8	64.8 ± 24.7	0.96 (0.93-0.98)	0.96 (0.94-0.99)

*adjusted by all variables in the table.

#### Reproducibility

Twenty-eight subjects completed the questionnaire twice. Reproducibility for the main question (occurrence of faecal impaction) was moderate, showing a 72% agreement and a weighted kappa of 0.46 (0.06-0.74). When only the subjects with no, or with mild cognitive impairment were selected (N = 25), reproducibility was good, with a simple agreement of 88% and a weighted kappa of 0.75 (0.43-1).

Among those with faecal impaction (N = 11), simple agreement was 54.5% for frequency, 81.8% for manual removal, 54.5% for retrograde lavage, and 45.5% for anterograde lavage.

#### Concurrent validity

Agreement between the self-reported occurrence of faecal impaction and the history recorded in medical records was fairly good, with a simple agreement of 85.9% and a weighted kappa of 0.72 (95% CI 0.62-0.82). None of the factors was associated with lack of agreement, as shown in Table 3, in the logistic regression model.

**Table 3:** Factors associated with lack of agreement (questionnaire vs medical records) with regard to the occurrence of faecal impaction in the last year

	**Agreement**	**Disagreement**	**Univariate OR**	Adjusted OR*
	**(N = 173)**	**(N = 25)**		
Age	86.2 ± 6.6	85.7 ± 7.1	0.99 (0.93-1.05)	0.98 (0.92-1.06)
Female Gender	124 /71.7%)	20 (80%)	1.58 (0.56-4.45)	1.56 (0.53-4.62)
Time at the institution	24.2 ± 24.0	28.3 ± 24.1	1.01 (0.99-1.02)	1.01 (0.99-1.02)
Educative level Secondary or higher	11 (6.4%)	0		
Cognitive impairment (MEC-35 score)	23.0 ± 8.5	22.6 ± 8.1	0.99 (0.95-1.04)	0.97 (0.91-1.03)
Functional impairment (Barthel’s score)	64.0 ± 29.0	69.0 ± 27.0	1.01 (0.99-1.02)	1.01 (0.99-1.03)
Use a proxy	140 (80.9%)	20 (80%)	0.75 (0.28-2.02)	0.51 (0.16-1.64)

*adjusted by all variables in the table.

## Discussion

Collecting information from the elderly is a difficult task, especially when self-reported information is required. Although some instruments to evaluate self-reported information on digestive symptoms have been specifically tested in the elderly [29], faecal impaction was not included; moreover, faecal impaction is likely to be associated with cognitive impairment [16], so any study on faecal impaction should take this limitation into account.

So far we did not have a validated questionnaire to collect information from fecal impaction, so studies were based on the information contained in the medical record or the patient’s response to a single question about fecal impaction history in recent months [30].

Our study shows that a simple questionnaire is reliable for collecting information on faecal impaction in the elderly and the limitations imposed by cognitive impairment are minimized by using a proxy.

Feasibility is one major concern with self-reported questionnaires in the elderly. The ability to understand the questions and answer them appropriately is markedly influenced by cognitive impairment [1] and, depending on the complexity of the questions [3], level of education. Our questionnaire is simple enough to be understood even by subjects with a primary level of education. Nonetheless, the feasibility is remarkably influenced, as would be expected, by cognitive impairment.

The best way to overcome the limitation imposed by cognitive impairment in the use of self-reported questionnaires is using the help of proxies. In general, proxies are considered useful and reliable [13]. In our study, we do not limit or direct the use of proxies, and subjects were allowed to choose whether they needed a proxy or not and who participated as their proxy. Obviously, not understanding all of the questions were a primary reason to elect to use the help of a proxy, but among those who did understand the questions, driving factors to choose a proxy were primary level of education, and cognitive and physical limitations. We have shown that the aid of a proxy overcomes the limitations inherent to our population, and specifically cognitive impairment. There is a fairly good agreement between subjects’ answers and medical and nursing records and, remarkably, lack of agreement is not related to education, cognitive impairment or the use of a proxy.

The main strength of our study is that the validation process was performed in a high quality of care nursing home since this is the best way to recruit a sample of elderly subjects with varying degrees of cognitive impairment. In fact, only subjects totally unable to complete the questionnaire were excluded, and a relevant proportion of the sample had moderate to severe cognitive impairment, allowing us to consider the questionnaire to be applicable for virtually all purposes. We should acknowledge some limitations. Firstly, we did not evaluate the agreement between self-reported and proxy-reported information. Since testing self-reported information against proxy-reported information is difficult and its value debatable [15], we tested the final effect directly, by comparing subject derived information (either self-reported or proxy-reported) to medical and nursing records. This can be considered a true gold standard in a nursing home, where all events occur inside the institution and are recorded, especially in a center like ours, with the highest external certifications of the quality of care and processes. Secondly, although reproducibility of the main question is fairly good, reproducibility of some items is relatively low, especially items regarding the use of anterograde and retrograde lavage. This may be due simply to difficulties inherent in discriminating whether they were used just for constipation (a usual event in nursing homes) or specifically for faecal impaction, as suggested by the high reproducibility of the question regarding manual removal, which is used only for faecal impaction.

## Conclusions

Our study shows that a simple questionnaire is reliable for collecting information on faecal impaction in the elderly and the limitations imposed by cognitive impairment are minimized by using a proxy.

## Competing interests

The authors declared that they have no competing interest.

## Authors’ contributions

ER is the principal investigator of the study and its guarantor. MB, MD-R, ALR, and ER contributed to the study design. MB and MJJ-C coordinated the recruitment of study participants and data collection. ER performed the statistical analysis. MB and ER drafted the manuscript. All authors contributed to data interpretation, revised the manuscript for important intellectual content, and approved the final version for publication.

## Pre-publication history

The pre-publication history for this paper can be accessed here:

http://www.biomedcentral.com/1471-2318/13/24/prepub
